# The Evolutionary Fate of the Horizontally Transferred Agrobacterial Mikimopine Synthase Gene in the Genera *Nicotiana* and *Linaria*

**DOI:** 10.1371/journal.pone.0113872

**Published:** 2014-11-24

**Authors:** Viera Kovacova, Jitka Zluvova, Bohuslav Janousek, Martina Talianova, Boris Vyskot

**Affiliations:** Department of Plant Developmental Genetics, Institute of Biophysics, Academy of Sciences of the Czech Republic, v.v.i., Kralovopolska 135, Brno, Czech Republic; Tel Aviv University, Israel

## Abstract

Few cases of spontaneously horizontally transferred bacterial genes into plant genomes have been described to date. The occurrence of horizontally transferred genes from the T-DNA of *Agrobacterium rhizogenes* into the plant genome has been reported in the genus *Nicotiana* and in the species *Linaria vulgaris*. Here we compare patterns of evolution in one of these genes (a gene encoding mikimopine synthase, *mis*) following three different events of horizontal gene transfer (HGT). As this gene plays an important role in *Agrobacterium*, and there are known cases showing that genes from pathogens can acquire plant protection function, we hypothesised that in at least some of the studied species we will find signs of selective pressures influencing *mis* sequence. The mikimopine synthase (*mis*) gene evolved in a different manner in the branch leading to *Nicotiana tabacum* and *N. tomentosiformis*, in the branch leading to *N. glauca* and in the genus *Linaria*. Our analyses of the genus *Linaria* suggest that the *mis* gene began to degenerate soon after the HGT. In contrast, in the case of *N. glauca*, the *mis* gene evolved under significant selective pressures. This suggests a possible role of mikimopine synthase in current *N. glauca* and its ancestor(s). In *N. tabacum* and *N. tomentosiformis*, the *mis* gene has a common frameshift mutation that disrupted its open reading frame. Interestingly, our results suggest that in spite of the frameshift, the *mis* gene could evolve under selective pressures. This sequence may still have some regulatory role at the RNA level as suggested by coverage of this sequence by small RNAs in *N. tabacum*.

## Introduction

Horizontal (lateral) gene transfer (HGT) is defined as a heritable change caused by transfer of genetic material between two species by non-sexual means. To remain widely open to HGT is a risky strategy of evolution that probably operates only in unicellular organisms. A horizontally transferred gene may be either harmful or beneficial and there is no certainty that the acquired gene increases the fitness of the individual as it has not been tested in parents [1]. HGT is a common and well-studied event in the microbial world and serves mainly for increasing the survival of organisms in changing conditions of the environment [2]. In prokaryotic organisms, a rapid gain of function is facilitated by a transfer of complete operons. HGT represents a driving force in the evolution of unicellular species where fixation of transferred material occurs straightforwardly in view of the fact that there is no separate germline as in multicellular organisms with differentiated tissues [3]–[5]. Interestingly, some mechanisms of protection against undesirable acquisition of genetic information exist even in prokaryotes, e.g., the mechanism using clustered regularly interspaced short palindromic repeats (CRISPR) and CRISPR associated (Cas) genes. This system was found in 40% of bacteria and 90% of archea [6],[7].

Gene transfer to multicellular organisms appears to be much rarer and more complicated than in the case of HGT between prokaryotes. In multicellular organisms, the complexity of eukaryotic organisms and their nuclei makes them more resistant to transfer of foreign DNA than in prokaryotes and unicellular eukaryotes. The number of reports of HGT in eukaryotic genomes is slowly accumulating. The detection of horizontally transferred sequences is, however, more complicated given that some of them are not expressed and cannot be found in RNAseq studies. Also in high throughput sequencing projects using DNA samples, sequences that could be obtained by HGT from bacteria are often filtered off as a possible contamination of the sample. In spite of these difficulties, rapid accumulation of a vast amount of sequencing data is uncovering the first evidence for HGT between prokaryotic and eukaryotic genomes and cases with actively expressed genes [8],[9]. Several examples of HGT between viruses and eukaryotes and between bacteria and eukaryotes have been found in economically important agricultural plant species (*Nicotiana* sp., *Lycopersicon* sp., and *Petunia* sp. [10]) and in intensively studied model species such as the nematode *Caenorhabditis elegans* [11] and the moss *Physcomitrella patens* [12]. A unique case is a massive uptake of foreign mitochondrial genes from a wide range of plant donors by a basal angiosperm, *Amborella trichopoda* [13].

Even in multicellular eukaryotes, HGT can enable the recipients to show new phenotypes that cannot be achieved by mutations or selection. This acceleration of evolution can confer huge advantage to adaptive processes or in speciation [14],[15]. Examples of HGT in the context of adaptive evolution include an HGT of multiple genes from bacteria and archaea into the red alga *Galdieria sulphuraria* that facilitates its evolution as an extremophile [5], a transfer of the bacterial gene *HhMAN1* coding mannanase to coffee berry borer beetles *Hypothenemus hampei* [16], a transfer of fungal genes coding carotenoid production into pea aphids (*Acyrthosiphon pisum*) genome [17], and a transfer of bacterial gene coding catalases and arsenite reductase into fungal genomes [18]. Purifying selection in horizontally transferred genes and the surprising direction of transmission of genetic material from eukaryotes (mosquitoes of the genus *Aedes*) to endosymbiotic bacteria *Wolbachia pipientis* was established in a gene family coding the salivary gland surface in mosquitoes [19].

A widely-spread method of human-controlled HGT is preparation of transgenic plants using *Agrobacterium* mediated transformation. Interestingly, there are also several examples of the transformation that occurred spontaneously, i.e., without a human activity. At least two transformation events by T-DNA coming from *A. rhizogenes* have been reported in the genus *Nicotiana* (in the clade leading to *N. glauca* and in the clade leading to *N. tomentosiformis* and *N. tabacum*; [20]). Recently, a spontaneous insertion of T-DNA from *A. rhizogenes* has also been reported in *Linaria vulgaris* [21]. The complex interactions between T-DNA carrying Agrobacteria and their plant hosts show how complicated systems can arise based on the host pathogen co-evolution. Both *A. tumefaciens* and *A. rhizogenes* are able (via transfer of the part of their plasmid DNA) to modify the metabolism of the host so that they induce production of plant hormones to stimulate excessive growth (*A. rhizogenes*: roots; *A. tumefaciens* tumors). Moreover, the bacteria enable plants to produce opines - nutrients that cannot be metabolized by the plant but by *Agrobacterium*. Most progress in the study of plant-*Agrobacterium* interaction was done in *A. tumefaciens*, a species closely related to *A. rhizogenes* [22]. In agrobacteria, opines also play a role in a quorum sensing signalization by stimulating the synthesis of N-3-oxo-octanoyl-homoserine lactone (OC8-HSL). In this way, opines stimulate multiplication of Ti plasmid in their cells and an exchange of genetic information between bacteria by conjugation [22]. Agrobacterial strains producing high levels of OC8-HSL are known to cause marked tumor growth. Plants have evolved a system enabling them to reduce production of OC8-HSL by bacteria. This is based on the synthesis of non-proteinogenic amino acid γ-aminobutyric acid (GABA), which stimulates degradation of OC8-HSL [23]. The plant defence can be, however, overcome by AbcR1, a small RNA that inhibits translation of mRNA coding periplasmic binding protein Atu2422 that is necessary for the uptake of GABA by *Agrobacterium*.

Ti-plasmid carrying strains of *A. tumefaciens* are selectively disadvantaged in comparison with purely saprophytic strains of the same species in the absence of opines but they acquire selection advantage if they are in the proximity of host synthesising appropriate opine [24],[25]. The opines can, therefore, dramatically influence the percentage of pathogenic *A. tumefaciens* bacteria in the population [24],[25]. It was found that there are periods when infectious strains are almost extinct from the studied local population [25]. Apart from agrobacteria and hairy-roots of recently attacked plants, the gene responsible for the synthesis of one opine (mikimopine) is also present in several plant species in the genera *Linaria* and *Nicotiana*. Several authors formulated a hypothesis that the genes transferred from pathogen to its host can be used by the host, e. g., in protection against pathogen. Here, we studied whether the sequences of mikimopine synthase from these species show any signs of purifying selection (as expected if the gene performs a function advantageous for the plant) or if these sequences show signs of degeneration. We have studied patterns of selection in the evolutionary history of the mikimopine synthase (*mis*) gene in all plant species where this gene was found. The advantage of this approach is that it can provide some indication of their function even if they are expressed in some specific tissue and/or just in specific developmental stage and/or under specific circumstances (e.g., in presence of pathogen). Albeit this approach does not enable elucidation of the exact role of a given sequence, it is possible to deduce whether the sequence could play a role at the protein level, RNA level or if it is non-functional.

## Materials and Methods

### Biological materials and sequence data

Plant and bacterial material used in this study is listed in Table S1. Nuclear DNA was extracted from leaves using the DNeasy Plant Kit (Qiagen). PCR was performed as described in Table S2. PCR products were gel-extracted using a Gel extraction kit (Qiagen) and cloned into the pDrive vector (PCR cloning kit, Qiagen). Plasmids and PCR products were sequenced in DNA Macrogen Europe (Netherlands). All the sequences used in this study (both the sequences obtained in this study and the sequences retrieved from the GenBank database; [26]) are listed in Table S3 (for *Linaria* phylogenetics), Table S4 (*mis* containing *Nicotiana* contigs) and Table S5 (*mis* homologues). Datasets containing *N. tabacum* small RNA reads (GSM717861, GSM717862, GSM1055737, GSM1055739, GSM1055740) were retrieved from NCBI Gene Expression Omnibus [27]. *N. tabacum* and *N. tomentosiformis* genomic contigs were retrieved from GenBank (whole genome shotgun sequencing projects: AWOJ01000000, AWOK01000000, AYMY01000000, ASAG01000000). The data for the analyses of transcription of long RNA in the roots of *N. tabacum* were retrieved from Sequence Read Archive at NCBI [28]. We used the datasets with accession numbers: SRX495526, SRX495527 and SRX495529. The tblastx was used to find outgroups for phylogenetic analyses in the *mis* gene. As an appropriate outgroup, we identified the homolog of *mis* gene from *Eutypa lata* and the genes *riorf22* plus *riorf23* the *mis* homologs from the non-T-DNA part of Ri plasmid.

### Sequence alignment and phylogenetic trees

Phylogenetic trees were constructed based either on the sequence fragment containing mikimopine synthase sequence and ORF14 (for analyses of the *mis* evolutionary pattern in PAML 4.7a, [29]) or on the sequences of several chloroplast (rpl32-trnL, trnS-trnG, trnL-trnF, trnK-matK) and nuclear genes (rDNA, AGT1, at103) (in both cases to the test of monophyletic origin of mikimopine synthase homologs in *Linaria*). Accession numbers of respective sequences are listed in Table S5 and Table S3). Alignment of sequences was performed using MAFFT version 6 [30] with default parameters and the resulting alignment was manually edited using SeaView 4.2.5 [31]. The phylogenetic trees were constructed using maximum likelihood, maximum parsimony and Bayesian inference. The optimal models of evolution for maximum likelihood based methods (*mis* gene homologues: PhyML3, [32]; partitioned nuclear and chloroplast datasets: GARLI 2 program, [33]), for Bayesian tree inference (*mis* gene homologues; MrBayes 3.2.2: [34],[35]) and for Bayesian dating (partitioned nuclear and chloroplast datasets; BEAST 1.7.5, [36]) were determined using jModelTest version 2.0 [37],[38] using second-order Akaike information criterion (AICc). These optimal models are listed in Table S6.

The maximum likelihood trees of *mis* gene homologues were reconstructed using PhyML3 [39]. The tree topologies were estimated using the approach BEST that estimates the phylogeny using both nearest neighbor interchange and subtree pruning and regrafting. The tree search was started from BioNJ tree and 500 random starting trees. The branch support was estimated using Shimodaira-Hasegawa-like approximate likelihood ratio test (SH-aLRT; [40]. In the case of the concatenated datasets (chloroplast and nuclear sequences), the maximum likelihood based phylogenetic analysis was carried out using GARLI 2 program [33]. The models estimated using jModelTest 2.0 were applied to particular partitions of the chloroplast and nuclear datasets. The majority rule consensus trees were constructed from the output of the bootstrapping analysis in SumTrees program (from DendroPy library, [41]).

In the case of the Bayesian tree inference in the nuclear dataset and the chloroplast dataset, the datasets were analysed as partitioned and the phylogenetic trees were constructed using MrBayes 3.2.2 [35] with mixed model across GTR space. The convergence of the chains was tested and the burn-in proportion was estimated using Tracer version 1.5 [42]. For the Bayesian dating of the most recent common ancestor of the species containing *mis*, we used calibration according to [43] so that the age of divergence between *Linaria* and *Antirrhinum* was constrained to 20±4 million years (based on the five Lamiales fossils and a divergence time between Oleaceae and Antirrhineae, modelled as a normal distribution with a mean 74 million years and Std = 2.5 million years; [43]). The models estimated using jModelTest 2 were applied to particular partitions of the chloroplast and nuclear datasets. The convergence of the chains was tested and the burn-in proportion was estimated using Tracer version 1.5 [42]. The obtained multi-tree files were summarized using TreeAnnotator (component of BEAST 1.7.5 package; [44]) into one Maximum credibility tree with median node heights. Trees were visualized using FigTree 1.4.0 [45]). To monitor the complexity of the species tree sets obtained in BEAST, we applied DensiTree 2.01 software [46].

Maximum parsimony analysis was carried out using PAUP* [47]. Heuristic search was performed with 1000 random addition sequence replicates using tree-bisection-reconnection branch swapping algorithm, the MULTREES option in effect. Gaps were treated as missing data. Node support was obtained using 5000 bootstrap replicates using the same heuristic search settings. The CONSEL package [48] was used to perform the approximately unbiased test [49] for putative congruence of the trees based on the chloroplast sequences and the nuclear dataset and vice versa. The tree topologies were compared using Compare2tree program [50].

### PAML analyses

PAML analyses were used to determine whether some of the copies of the *mis* gene evolved under selective pressure. The CODEML program of PAML 4.7a [29] was used to estimate the ratio (*ω*) of the non-synonymous substitution rate (*d_N_*) to the synonymous substitution rate (*d_S_*). In the *mis* alignment, we removed frameshift insertions and recoded the stop codons as missing data as, e.g., performed by others [51]–[53].

As the reference tree, a modified maximum likelihood phylogenetic tree based on the alignment of mikimopine synthase sequences was used. In the original maximum likelihood tree, sequences of the left and right copies of *N. glauca* did not form a clade rather than a grade (*mis* in *N. glauca* is arranged as inverted repeat). Because the copies were named “left” and “right” both in *N. glauca* and *Linaria vulgaris*, we continue to use this nomenclature. Since both these copies apparently come from one insertion event [20] and because this topology is supported only by six parsimony-informative sites (the topology where all *N. glauca* sequences form a clade is supported by four parsimony-informative sites), we constructed the modified tree where the positions of *N. tomentosiformis* plus *N. tabacum* and of both copies of *N. glauca* are unresolved. Given that the sequences obtained in the majority of the studied *N. tabacum* accessions and in *N. tomentosiformis* are identical, we retained just one left and one right copy of *N. tabacum* and *N. tomentosiformis* to avoid bias in favour of substitutions characteristic for this section.

The maximum likelihood tree based on the sequences of nuclear genes was also used in *Linaria* as a reference tree to analyse the concatenated coding sequences of the genes At103, rpl32 and matK to exclude/or confirm the role of low effective population size in relaxation of selection constraints observed in the *mis* gene.

The equilibrium frequencies of codons were calculated from the nucleotide frequencies (CodonFreq = 2) because it best fits the data as calculated by second-order AIC. To substantially decrease the computation time demand, the codon-based branch lengths were estimated under the one-ratio model (M0) and the resulting tree with estimated branch lengths was used for the modelling of other models. Modelling of all models was performed with three initial ω values: ω = 0.5, ω = 1, and ω = 2.

Both branch and branch-site models were applied to (i) branches leading to the most recent common ancestor (MRCA) of all *Linaria* species, to the MRCA of *Nicotiana glauca*, to the MRCA of *Nicotiana tabacum* plus *N. tomentosiformis* (all these branches correspond to the situation before the insertion of *mis*), and to (ii) branches corresponding to the inserted *mis*. In the branch analyses, two-ratios models were compared to three-ratios models to reveal whether the evolutionary pattern of *mis* before its insertion is significantly different from the evolutionary pattern of *mis* after its insertion. In the branch-site analyses, modified model A was compared with both the corresponding null model with *ω*_2_ = 1 fixed (test 2; the null distribution is the 50∶50 mixture of point mass 0 and χ^2^_1_) and with the model M1a (test 1; [54]).

The resulting log likelihood values were evaluated using likelihood-ratio tests to determine any statistical significance of the difference. The chi2 program of PAML was used to estimate the P-values. The confidence intervals for proportion were computed online (http://vassarstats.net/prop1.html) using the method with continuity correction [55] that is derived from a procedure outlined in [56].

### RNA read mapping

The small RNA reads were mapped using the Galaxy [57]–[59] version of LASTZ alignment algorithm [60]. The parameters were adjusted disallowing mismatches. As query sequences, the *mis* sequence coming from *N. tabacum var. chinensis*, a 23 kbp long *N. tabacum* contig containing *mis* (AWOK01262755) or a set of 100 randomly chosen *N. tabacum* genes retrieved from the European Nucleotide Archive were chosen (for the accession numbers see supplementary File S1). In order to compare the number of reads per million between genes, the numbers of reads were normalized based on the gene length. Long RNA based reads were mapped to reference (AWOK01262755) using Bowtie [61] implemented in RSEM [62]. The transcript quantification was performed in RSEM [62]. We have used the algorithm for paired-end RNA-Seq data.

### Genetic transformation experiment

We used aseptically grown seedlings of the *Nicotiana tabacum* (cv. Vielblattriger) and the strains of *A. rhizogenes* containing T-DNA with *mis* (MAFF0210265, MAFF0210266, MAFF0210267, MAFF0210269, and MAFF0301725) and *A. rhizogenes* strain A4RSII (rifampicin and spectinomycine resistant variant of A4 strain, agropine type of T-DNA, reviewed by [63]). The A4RSII strain has been routinely used in our laboratory to induce hairy-roots in wide spectrum of hosts (e.g., tobacco, *Rumex acetosa*, *R. acetosella*, *Silene vulgaris*, and *S. latifolia*). The ability to promote hairy-roots in *N. glutinosa* (species that does not contain mikimopine type T-DNA insertion, [64]) was previously reported in most of the tested strains (MAFF021265, MAFF0210266, MAFF2110267 and MAFF210269) [65]. Strain MAFF0210265 induced hairy roots in *N. glutinosa*, tomato and peanut and the strain MAFF0210266 induced hairy roots in *N. glutinosa*, tomato, peanut and cucumber [65]. The leaf discs (about 50 leave discs for each *A. rhizogenes* strain; size approx. 1 cm^2^) were treated with suspension of bacteria in LB medium for 15 min and then cultivated on BMS-30 medium for 48 hrs [66]. Negative control samples were incubated in LB medium only and then cultured identically as other samples. Positive control samples were treated using the *A. rhizogenes* strain A4RSII that does not contain the *mis* synthase gene.

## Results

### *N. tabacum* and *N. tomentosiformis* contigs containing mikimopine synthase

We used the BLAST search to identify contigs containing sequences homologous to mikimopine synthase in recently published data in *N. tabacum* [67] and *N. tomentosiformis* [68]. In both *N. tabacum* and *N. tomentosiformis*, we found two different sequences homologous to *mis:* a sequence showing 99% similarity to the previously published *N. tabacum mis* (FN667970.1; [69]), hereafter referred to as *mis1*, and a newly identified sequence with 65% similarity to *N. tabacum mis*, hereafter referred to as *mis2*. The contings containing *mis* homologs are listed in the Table S4. Both *mis1* and *mis2* are arranged as inverted repeats. Interestingly, a duplication of T-DNA sequences containing *mis* was also described in *N. glauca* as inverted repeat [69]) and in *Linaria vulgaris* as direct repeat [70].

**Figure 1:**
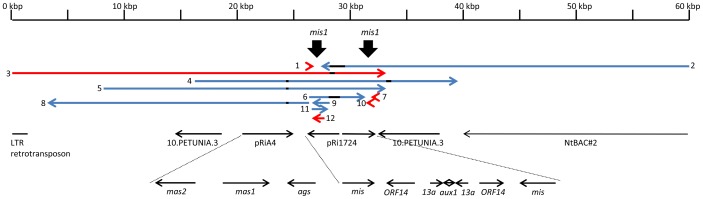
Scheme of position of *N.tabacum* contigs containing *mis1*. *Nicotiana tomentosiformis* contigs are given in red, *N. tabacum* contigs are blue,gaps within contigs are black. Bold black arrows point to the *mis*1 sequences. Black arrows under the scheme of contigs show position and orientation of sequences homologous to the contigs: 1: ASAG01208860, 2: AYMY01187867, 3: ASAG01039652, 4: AWOK01262755, 5: AWOJ01062996, 6: AWOJ01534161, 7: ASAG01205334, 8AYMY01187868,9-AYMY01393623,10-ASAG01208934,11-AWOK01658345, and 12-ASAG01214370.

As shown in Figure 1, the pRi containing *mis1* is both in *N. tomentosiformis* and *N. tabacum* inserted within the *Nicotiana* homolog of the gene *10.PETUNIA.3*. The region originating from the pRi insertion is formed of incomplete inverted repeat. The left part of the region carries homologues of mannopine and agropine synthase genes *mas2*, *mas1* and *ags* that are present both in *A. rhizogenes* and *A. tumefaciens*. The inverted repeat carries *mis1*, *ORF14*, *ORF13a* and a part of *aux1* (Figure 1) that are exclusively present in *A. rhizogenes*. The *mis2* region is formed both in *N. tabacum* and *N. tomentosiformis* by inverted repeats. The length of the regions that are present between *mis2* copies markedly differs in respective *N. tabacum* accessions (Figure S1). In contrast to the *mis1* region, we were not able to identify surrounding sequences, because the contigs containing *mis2* were very short.

### Phylogenetic analyses

**Figure 2:**
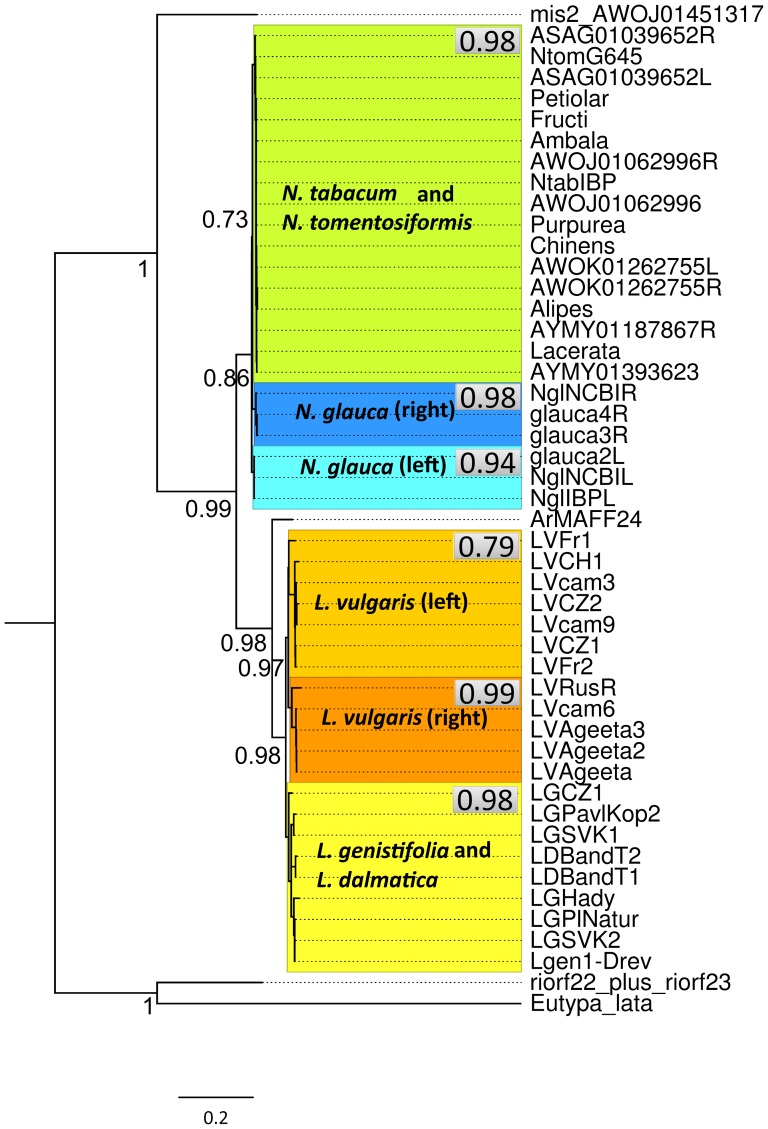
Maximum-likelihood analyses of the region containing gene for mikimopine synthase and the ORF14 gene *in A. rhizogenes* and their plant homologues in *N. glauca*. The clades belonging to individual plant species are marked in colour. If left and right copy were present they are also distinguished by colour. The support values (aLRT) are not shown for the nodes inside the marked blocks. Support value for each marked block is shown inside the grey rectangle in the right upper corner of this block. For the support values and the topology inside the marked blocks see Figure S2A.

We reconstructed the phylogeny of horizontally transferred T-DNA region in the genera *Nicotiana* and *Linaria* based on the fragment including both *mis* and ORF14 sequences. These analyses were done using maximum likelihood (Figure 2, Figure S2A,B) and Bayesian methods (Figure S2C). In spite of the previously proven independent origin of T-DNA insertion in the common ancestor of *N. tomentosiformis* and *N. tabacum*, and in *N. glauca* [20], sequences of these three species grouped together. This could be caused by differences in *Agrobacterium* strains attacking *Linaria* and *Nicotiana*, i. e., there could be a common ancestor of all strains of *A. rhizogenes* that attacked the genus *Nicotiana*; and this ancestor had diverged from the common ancestor of strains attacking *Linaria*. Two copies of *mis* arranged as direct repeats in *L. vulgaris* [70] have been described but no information was available on *mis* in *L. genistifolia* or *L. dalmatica*. The phylogenetic tree based on the sequences of *mis* and *ORF14* indicates that the horizontally transferred sequence was duplicated after *L. vulgaris* diverged from *L. genistifolia* and *L. dalmatica*.

**Figure 3:**
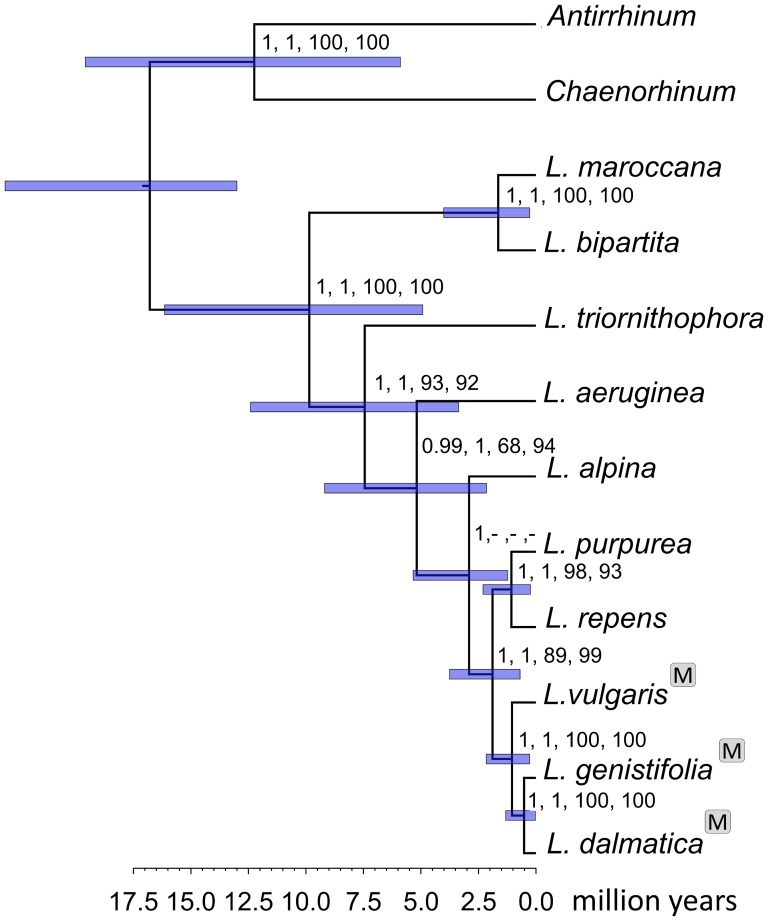
Test of the monophyletic origin of *mis* insertion in the genus *Linaria*. The chronogram is based on the nuclear sequence dataset. The species carrying the mikimopine synthase gene are labelled by the M letters in a grey box. The support values for each node are listed as follows: posterior probability obtained in Beast 1.7.5, posterior probability obtained in MrBayes 3.2, bootstrap value obtained in GARLI 2.0, and bootstrap value obtained in PAUP 4.0. (The posterior probabilities are not shown if they are lower than 0.8 and the bootstrap values are not shown if they are lower than 60.) The horizontal bars represent 95% HPD (highest posterior density) intervals of node ages in million years.

To test the monophyletic origin of T-DNA insertion in the genus *Linaria*, we performed a phylogenetic analysis based on the datasets of nuclear and chloroplast genes (Figure 3 and Figure S3A–D). The trees of the genus *Linaria* differed slightly depending on the dataset from which they were inferred (nuclear or chloroplast). The difference between the datasets is supported by the results of the approximately unbiased test which show that the chloroplast dataset based tree (obtained via maximum likelihood method) does not fit the nuclear dataset (P = 0.031). The nuclear dataset based tree (obtained via maximum likelihood method) also does not fit with the chloroplast data (P = 5×10^−5^). As apparent from the comparison of both topologies (Figure S3D), the difference is caused by a different position of *L. alpina* and *L. aeruginea* (both from the section Supinae). The multi-tree file generated by BEAST 1.7.5 based on the nuclear dataset did not show any conflicting signal when analysed by DensiTree 2.0.1 [46] (Figure S4A). In the chloroplast dataset, the heterogeneity of trees in Beast 1.7.5 multi-tree output file is apparent when visualized in DensiTree 2.0.1 [46] (see Figure S4B). In spite of this difference, the distribution of the T-DNA carrying species in the phylogenetic trees of the *Linaria* genus unequivocally shows that the origin of the *mis* sequences in species of the genus *Linaria* is monophyletic (Figure 3, Figure S3) as they are present in only three species of monophyletic origin (*L. vulgaris*, *L. genistifolia* and *L. dalmatica*). As expected, provided the monophyletic origin of *mis* is assumed, the phylogeny of the nuclear and chloroplast sequences of these three species showed the same topology as in the case of the *mis* gene homologs (Figure S2). The estimated median of HPD for the age of the most recent common ancestor of the species carrying the *mis* gene obtained in BEAST 1.7.5, was both in nuclear and chloroplast datasets approximately 1 million years (Figure 3, Figure S3A).

### Estimation of the pattern of evolution using PAML

To determine whether some of the copies of the *mis* gene evolved under selective pressure, we applied branch and branch-site models to the *mis*-ORF14 T-DNA fragment-based phylogenetic tree that showed independent insertion of the T-DNA into *N. glauca* and into *N. tabacum* plus *N. tomentosiformis*. The branch analysis of the branch before and branches after the *mis* insertion in the genus *Linaria* revealed that the ω value before the insertion of *mis* is significantly lower than after the *mis* insertion (Table 1). The branch-site analysis of the internal branch shows that approximately 73% of codons are consistent with purifying selection. On the other hand, the analysis of *Linaria* terminal branches revealed the effects of purifying selection in approximately 10% of codons, while 90% of codons evolved neutrally (in 86% of codons, relaxed selective constrains were detected). This decrease in codons under purifying selection during the evolution of the *mis* gene in the genus *Linaria* is statistically significant (Table 2). The hypothesis of degeneration of this gene is further supported by the presence of frameshift mutations that lead to numerous stop codons (Table S5). We also performed a separate analysis in *L. vulgaris* and *L. genistifolia* that showed decrease in codons under purifying selection in both these species (Table S7). The decrease in codons under purifying selection is probably not caused by a low effective population size of *L. genistifolia* because the decrease in percentage of codons under purifying selection was also found in *L. vulgaris* which is a widespread species (Table S7). In contrast to the *mis* gene, the branch-site analysis of one nuclear and two chloroplast genes did not show any decrease in percentage of the codons under purifying selection in terminal branches (Table S8). This result supports the view that *L. genistifolia* is not affected by low effective population size.

**Table 1:** Branch analysis of mikimopine synthase evolution before and after HGT.

	branches		
host	ω before HGT	ω after HGT	P
genus *Linaria*	0.40	0.80	2.73×10^−9^
*Nicotiana glauca*	0.31	1.04	0.13
*N. tabacum* and *N. tomentosiformis*	0.31	0.53	1

P-values after Bonferroni correction show level of statistical significance for hypothesis that there are no differences. Highly significant difference was found in *Linaria*.

**Table 2:** Branch-site analysis of mikimopine synthase evolution before and after HGT.

		codons consistent with							
		ω<1		ω = 1		ω>1		tests	
host	branches	percentage	95% CI (%)	percentage	95% CI (%)	percentage	95% CI (%)	test 1	test 2
genus *Linaria*	internal	**73.10%**	67.98–77.73	**26.90%**	22.27–32.02	0.00%	0–1.41	1	1
	terminal	**10.06%**	7.23–14.02	**89.94%**	85.98–92.77	0.00%	0–1.41	5.96×10^−3^	0.12
*Nicotiana glauca*	internal	73.10%	67.98–77.73	26.91%	22.27–32.02	**0.00%**	**0–1.41**	1	1
	terminal	68.67%	63.35–73.53	24.57%	20.04–29.51	**6.76%**	**4.5–10.27**	5.96×10^−3^	0.012
*N. tabacum and N. tomentosiformis*	internal	73.10%	67.98–77.73	26.91%	23.27–32.02	0.00%	0–1.41	1	1
	terminal	70.84%	65.51–75.50	26.73%	21.99–31.70	2.44%	1.12–4.84	1	0.99

Tests 1 and 2 are explained more in detail in Materials and methods. P-values after Bonferroni correction. CI - confidence interval, statistically significant difference between the internal and terminal branches are marked in bold.

In *N. glauca* and *N. tabacum* plus *N. tomentosiformis*, the analyses showed no statistically significant differences between internal and terminal branches in terms of the ω values calculated by branch models (Table 1) or between internal and terminal branches in percentage of codons under purifying selection (and under neutral evolution) as calculated by branch-site models (Table 2). Likelihood ratio tests did not exclude purifying selection in either *N. glauca* or in *N. tomentosiformis* plus *N. tabacum*. We also detected a low fraction of codons with ω>1 in the terminal branches. In *N. glauca*, all the sequences analysed retained intact open reading frame without any frameshift mutation. On the other hand, all sequences of *N. tabacum* and *N. tomentosiformis* have common frameshift mutation in the nucleotide position 139 that leads to numerous stop codons (Table S5).

### Analysis of small RNAs complementary to *mis* in *N. tabacum* and transformation experiments

**Figure 4:**
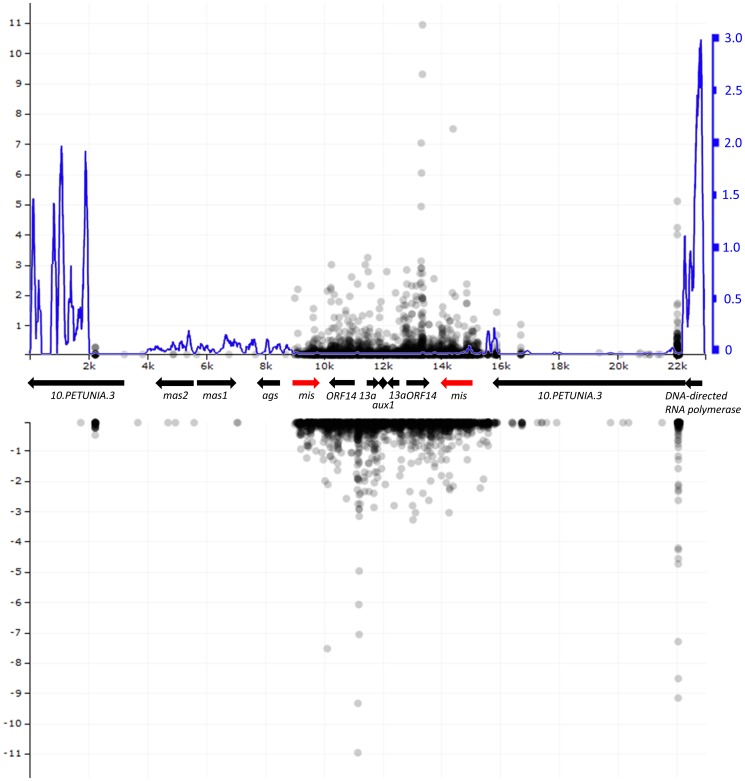
Analysis of the distribution of small RNAs and long RNAs along the contig AWOK01262755 containing two copies of *mis1*. These analyses were performed with dataset based on root samples. A. Distribution of small RNAs mapped to the upper strand of the contig (as dots in black) and long RNA based reads in both strands (line in blue). The x-axis shows the distance from beginning in kb. The y-axis on left (in black) shows the number of reads of small RNAs per milion. The y-axis on the right (in blue) shows number of the long RNA based reads per million. B) Distribution of small RNAs (as dots in black) mapped to the bottom strand of the contig. The y-axis on left (in black) shows the number of reads of small RNAs per million. Negative values are used to stress that the reads were mapped to the bottom strand. In order to keep suitable size of the figure, four values are not displayed at the bottom strand (22.028 kb: −111, 22.034 kb: −68, 22.035 kb: −23, and 22.029 kb: −21). The arrows between the two graphs show detected homology to known sequences. The two *mis1* copies are in red. Note the accumulation of the small RNA reads in the region including two copies of *mis1*. In contrast, the region including *mis1* copies shows just very low abundance of the long RNA based reads.

We were not able to disclose purifying selection of *mis* either in *N. tabacum* or in *N. tomentosiformis*, although *mis* was disrupted in both these species by the common frameshift mutation causing numerous stop codons. The most likely explanation is that *mis* could act at the RNA level. To test this hypothesis, we mapped small RNA sequencing data to the *mis* sequence of *N. tabacum*. Codons displaying different evolutionary pattern were covered randomly in three different samples (root, stems and leaves) both in positive and negative strand (Table S9). As shown in Figure S5, the whole sequence was densely covered by small RNA molecules in all tissues examined. To compare the small RNA coverage of *mis* with other *N. tabacum* genes, we mapped small RNA sequencing data from roots to 100 randomly chosen coding sequences. The results are summarized in Figure S6. All analysed *N. tabacum* genes are less covered by small RNA reads than *mis*: the range of the genes analysed was 0.1–70.5 small RNA reads per one kilobase and for a million reads, the median was 0.98. In *mis*, 126 reads per kilobase and million reads were mapped in roots. Moreover, we mapped small RNAs from roots to the 23 kbp long contig AWOK01262755 that contains both the whole inverted repeat and their surrounding sequences (Figure 4). Apparently, the whole inverted repeat is densely covered by small RNA molecules, in contrast to its surrounding (Figure 4). A contrasting abundance pattern was found if reads obtained from long RNAs were mapped to the AWOK01262755 contig (Figure 4). Only very weak transcription was detected in the *mis* homologs (reads: SRR1199123.10005576, SRR1199123.1275854, SRR1199123.24804835, SRR1199123.28081512, SRR1199123.30550315, SRR1199123.42702794, and SRR1199122.8844895). The most likely explanation is that the *Nicotiana mis* copy generates numerous small RNA molecules that serve as defence against *A. rhizogenes* strains containing the *mis* gene. To test this hypothesis, we infected *N. tabacum* leaf discs with *A. rhizogenes* strains containing the *mis* gene (MAFF0210265, MAFF0210266, MAFF0210267, MAFF0210269, and MAFF0301725). No hairy roots were observed after four weeks of cultivation of leaf discs on the BMS-30 medium in a negative control and in all the *A. rhizogenes* strains containing the *mis* gene. In contrast, several hairy roots per explant (approximately five on average) were observed in a positive control (the strain without *mis* gene - A4RSII).

## Discussion

In principle, any horizontally transferred DNA sequence can either degenerate, be retained “as is” or undergo adaptive evolution. We compared the evolutionary fate of the mikimopine synthase gene after three different events of HGT. Analysis of the synonymous and non-synonymous substitutions showed that the *mis* gene evolved in a different manner in the branch leading to *N. tabacum* plus *N. tomentosiformis*, in the branch leading to *N. glauca*, and in the genus *Linaria*.

In the genus *Linaria*, the positions of the species containing T-DNA insertion in the phylogenetic tree (Figure 3, Figures S3 and S4) suggest that the HGT probably happened more than 1 million years ago (the median of HPD for the age of the most recent common ancestor of the species carrying the *mis* gene, Figure 3, Figure S3A). Our estimate of the age of the most recent common ancestor of *L. vulgaris*, *L. dalmatica* and *L. genistifolia* is in accordance with the estimates reported by other authors [43],[71]. Since the *Linaria mis* is degenerated in all species studied, it is likely that the *mis* sequence started to degenerate soon after the HGT. Therefore, it is possible to conclude that in spite of the step-wise decay of the horizontally transferred sequences that do not provide sufficient advantage to the host, these sequences can persist in the genome for a long time. The rarity of the naturally transformed species is therefore caused by the rarity of HGT events rather than by a loss of the horizontally transferred T-DNA sequences from the host species. The presence of the sequence without any function and fixed during speciation can be explained either as a consequence of low effective population size or as a result of hitchhiking of this gene by other gene(s) from T-DNA. These genes could be advantageous for the host (e.g., enhanced stress tolerance and suppression of reactive oxygen species as observed in *Rubia cordifolia* transformed by T-DNA *rolC* gene; [72]). Alternatively, a hitchhiking of the whole T-DNA by some other sequence present in the *Linaria* genome in the neighbourhood of T-DNA insertion could cause fixation of the T-DNA sequence in the population.

In contrast, both copies of the *mis* gene evolved under significant selective pressures in *N. glauca*. This result strongly suggests a possible function of mikimopine synthase in the current *N. glauca* and in its ancestor(s). Indeed, a certain level of transcription of both left and right *mis* copies has been detected in this species using RT PCR [20]. The right copy of the *mis* gene was tested for its ability to produce mikimopine when transferred to *Escherichia coli* and the result of this study was positive [73]. The presence of mikimopine can be advantageous for *N. glauca* in several ways. (i) There are signs of the possible role of opines in the increase of stress response in the T-DNA transformed *Solanaceae* [74]. Sauerwein and Wink (1993) tested a chemically synthesized mikimopine for its influence on alkaloid production in root cultures (both transgenic and non-transgenic) of *Hyoscyamus albus* and they found a positive effect on production of alkaloids that play a role in the defence mechanism of this species. (ii) Reduction of growth of larvae *Manduca sexta* was observed in opine-treated plants of *H. albus*. We can thus speculate that the *mis* gene expression could be increased during stress in particular tissues as a mediator of better stress response. (iii) Mikimopine also shows allelopathic effects - inhibition of germination was observed after a mikimopine treatment of seeds in *Lepidium sativum* [74]. Opine-producing plants alter their biological environment and in this way they modify rhizobacterial populations. Mutually advantageous relations to some bacterial species can be, therefore, established [75],[76]. (iv) Possible symbiotic relationships between some *Agrobacterium* species and their host plants have been also discussed [74]. Horizontally transferred genes for opines can provide to plants similar advantages if the function of these genes is retained.

Despite the fact that the *mis* gene has a two-nucleotide deletions causing numerous stop codons both in *N. tabacum* and *N. tomentosiformis*, more than 70% of its codons still evolves under purifying selection. In *N. tabacum*, it is possible to estimate the age of the horizontal transfer of *A. rhizogenes* T-DNA (including the studied *mis1* gene) based on the current estimate of the age of origin of *N. tabacum* from parental species [77]–[80] and the high similarity of *N. tabacum* and *N. tomentosiformis* sequences. These data suggest that this HGT event happened 0.01–0.2 million years ago. As all the samples of *N. tabacum* and *N. tomentosiformis* studied here contain the specific frameshift mutation, we can conclude that this mutation was present in their common ancestor. Thus, the *mis* gene has been evolving under purifying selection for more than ten thousand years, although it already possessed this frameshift mutation. Although it probably has no function at the protein level, the sequence can still have a function at the RNA level. The search for the small RNA molecules showing homology to the *mis* gene in *N. tabacum* has revealed dense coverage of this sequence by small RNAs (Figure 4 and Figure S5). Mikimopine synthase is covered by small RNAs densely – we have identified 128× more small RNA reads complementary to *mis* than to a median gene from our 100-genes dataset. Thus, it seems likely that *mis* was retained in *N. tabacum* and *N. tomentosiformis* for more than 10 thousand years with the above-mentioned frameshift mutation because it functions at the RNA level as a defence against *Agrobacterium* strains containing the *mis* gene. This hypothesis is supported by the fact that experimentally introduced RNAis against some T-DNA genes provoked resistance to tumorigenesis induced by *A. tumefaciens* [81]. The utilisation of the sequences acquired from pathogenic organism for protection of the host resembles the CRISPR/Cas system that works in prokaryotic organisms [6],[7] but so far no specific accumulation of small “samples” of DNA from pathogens has been described in plants.

The putative role of the horizontally transferred *mis* homolog in plant protection in *N. tabacum* is indirectly supported by the fact that no T-DNA carrying transformants of *N. tabacum* were obtained when several strains of *A. rhizogenes* (listed in Table S1) containing the *mis* gene were used in infection experiments. Based on the current knowledge of the role of small RNAs in interactions between plant hosts and bacteria [82], we can speculate on the influence of naturally occurring small RNAs produced from the *mis* gene in *N. tabacum*, which could block a further infection event by the mikimopine-type of *A. rhizogenes*. The putative mechanism may be based on endogenous siRNAs derived from the horizontally transferred *mis* gene or the *mis* gene transcripts may serve as permanent activator(s) of defence mechanisms. In the absence of a pathogen, the mechanisms for silencing unwanted sequences coming from pathogens are otherwise suppressed via small RNAs to be cost-effective (reviewed in [83]). The drawback of the necessity to activate the defence response via small RNAs is that bacteria are able to develop mechanisms that suppress the defence response at the very beginning (reviewed in [83]). It has been shown that suppression of the silencing mechanisms is necessary for a successful infection by *A. tumefaciens* and crown gall development [84]. Under some circumstances, it can be, therefore, advantageous for the host to maintain the defence mechanism in a stand-by status. Alternatively, the *mis* sequence may have been recognized as a junk sequence and its status may be controlled by a mechanism involving hc-siRNA (for a review see [85]). Interestingly, the host could acquire resistance to *Agrobacterium* even in this case. The bacterial sequence entering a host plant will be recognized by the machinery involving hc-siRNA and it will be methylated, in this case. At the population level, we can anticipate that the decreased or inhibited synthesis of mikimopine in *N. tabacum* also causes a decrease in representation of the pathogenic strains of *A. rhizogenes* in the local population in contrast to saprophytic strains.

## Supporting Information

Figure S1 **Scheme of position of** ***N. tabacum*** **and** ***N. tomentosiformis*** **contigs containing** ***mis2*** **.** *N. tomentosiformis* contigs are given in red, N. tabacum contigs are blue, gaps within contigs are black. Black arrows under the scheme of contigs show position and orientation of mis2 sequences. 1 - AWOJ01451316, 2 - ASAG01121015, 3 - ASAG01208987, 4 - AYMY01382493, 5 - AWOJ01451317, 6 - AWOJ01507417, 7 - AYMY01401705, 8 - AYMY01391287.
(PDF)

Figure S2 **Bayesian and maximum likelihood phylogenetic analysis of the gene for mikimopine synthase and the ORF14 gene in** ***A. rhizogenes*** **and their plant homologues in the genera** ***Nicotiana*** **and** ***Linaria*** **.** The clades belonging to individual plant species are marked in colour. If left and right copy are present they are also distinguished by colour. A) Maximum likelihood based tree displayed as cladogram to better show support values. B) Maximum likelihood based tree obtained with the reduced dataset (this tree was used for the PAML calculations). C) Bayesian analysis on the complete dataset.
(PDF)

Figure S3 **Tests of the monophyletic origin of the** ***mis*** **insertion in the genome of Linaria and dating of this HGT event.** A) The chronogram is based on the analysis of the chloroplast sequence dataset in Beast 1.7.5. The species carrying the mikimopine synthase gene are labelled by the M letters in a grey box. The support values for each node are listed as follows: posterior probability obtained in Beast 1.7.5, posterior probability obtained in MrBayes 3.2, bootstrap value obtained in GARLI 2.0, and bootstrap value obtained in PAUP 4.0. (The posterior probabilities are not shown if they are lower than 0.8 and the bootstrap values are not shown if they are lower than 60.) The horizontal bars represent 95% HPD (highest posterior density) intervals of node ages in million years. B) Phylogenetic tree obtained via maximum likelihood method (GARLI) using nuclear dataset in the genus Linaria. C) Phylogenetic tree obtained via maximum likelihood method (GARLI) using chloroplast dataset in the genus Linaria. D) Comparison of the phylogenetic trees obtained via maximum likelihood method (GARLI) using nuclear or chloroplast dataset using Compare2tree program.
(PDF)

Figure S4 **Analysis of the putative heterogeneity of the trees sampled in Beast analysis.** First 25% trees were removed as burn in. The most frequent topologies and the corresponding consensus trees are shown in blue. The second most frequent topology is shown in red and the corresponding consensus tree is shown in green. A) nuclear dataset – analysis of 20000 trees from Beast 1.7.5 output in DensiTree 2.0.1. B) chloroplast dataset – analysis of 20000 trees from Beast 1.7.5 output in DensiTree 2.0.1.
(PDF)

Figure S5 **Analyses of the distribution of small RNAs along the** ***mis*** **sequence homolog in N. tabacum.** The abundance of reads is shown as number of reads per one kilobase and per million of reads.
(PDF)

Figure S6 **Analysis of the abundance of homologous small RNAs for 100 randomly chosen genes in** ***N. tabacum*** **.** The abundance of reads is shown as number of reads per one kilobase and per million of reads These results were compared with results obtained in *mis*.
(PDF)

Table S1 **List of plant and bacterial material used for PCR detection of** ***mis*** **genes and** ***mis*** **homologs and/or for the genetic transformation experiment.**
(XLSX)

Table S2 **PCR primers and PCR profiles used in this study.**
(XLSX)

Table S3 **List of the sequences in the concatenated nuclear and chloroplast datasets used for phylogenetic analyses including vouchers and accession numbers.**
(XLSX)

Table S4 **List of the** ***N. tomentosiformis*** **and** ***N. tabacum*** **contigs that show homology to** ***N. tabacum mis*** **.**
(XLSX)

Table S5 **Number of frameshift mutations and stop codons in** ***mis*** **of analysed plants** (accession numbers included).
(XLSX)

Table S6 **Nucleotide substitution models optimal for particular sequences as identified using jModelTest.**
(XLSX)

Table S7 **Branch-site analysis of the** ***mis*** **gene in the genus** ***Linaria*** **.** Both *L. genistifolia* and *L. vulgaris* show significant decrease of codons under purifying selection when compared with the internal branch leading to the genus *Linaria*. CI - confidence interval.
(XLSX)

Table S8 **Branch-site analysis of one nuclear and two chloroplast genes in the genus Linaria.** Neither *L. genistifolia* nor *L. vulgaris* show significant decrease of codons under purifying selection when compared with the internal branch leading to the genus Linaria. CI - confidence interval.
(XLSX)

Table S9 **P-values of Fisher exact test testing independent distribution of small RNA molecules with respect to the codons under different under different evolutionary mode.** The results clearly show that the distribution of small RNAs is random.
(XLSX)

File S1 **Multi-fasta file containing sequences of the 100 randomly chosen genes used for the analysis of the abundance of homologous small RNAs in** ***N. tabacum*** **(see Fig. S6).**
(TXT)
