# Supplementary material for: The Evolutionary Fate of the Horizontally Transferred Agrobacterial Mikimopine Synthase Gene in the Genera Nicotiana and Linaria
Source: PLoS One. 2014 Nov 24;9(11):e113872. doi: 10.1371/journal.pone.0113872 (PMC4242671; doi:10.1371/journal.pone.0113872)
Supplement: Figure S1 — Scheme of position of N. tabacum and N. tomentosiformis contigs containing mis2 . N. tomentosiformis contigs are given in red, N. tabacum contigs are blue, gaps within contigs are black. Black arrows under the scheme of contigs show position and orientation of mis2 sequences. 1 - AWOJ01451316, 2 - ASAG01121015, 3 - ASAG01208987, 4 - AYMY01382493, 5 - AWOJ01451317, 6 - AWOJ01507417, 7 - AYMY01401705, 8 - AYMY01391287. (PDF) [file pone.0113872.s001.pdf]

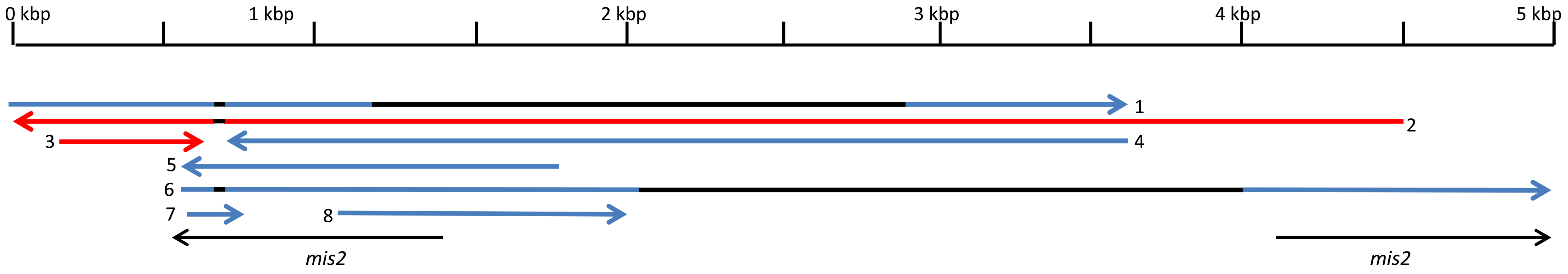

Figure S1. Scheme of position of *N. tabacum* and *N. tomentosiformis* contigs containing *mis2*. *N. tomentosiformis* contigs are given in red, *N. tabacum* contigs are blue; gaps within contigs are black. Black arrows under the scheme of contigs show position and orientation of mis2 sequences: 1-AWOJ01451316, 2-ASAG01121015, 3-ASAG01208987, 4-AYMY01382493, 5-AWOJ01451317, 6-AWOJ01507417, 7-AYMY01401705, and 8-AYMY01391287.
