# Supplementary material for: The Evolutionary Fate of the Horizontally Transferred Agrobacterial Mikimopine Synthase Gene in the Genera Nicotiana and Linaria
Source: PLoS One. 2014 Nov 24;9(11):e113872. doi: 10.1371/journal.pone.0113872 (PMC4242671; doi:10.1371/journal.pone.0113872)
Supplement: Figure S2 — Bayesian and maximum likelihood phylogenetic analysis of the gene for mikimopine synthase and the ORF14 gene in A. rhizogenes and their plant homologues in the genera Nicotiana and Linaria . The clades belonging to individual plant species are marked in colour. If left and right copy are present they are also distinguished by colour. A) Maximum likelihood based tree displayed as cladogram to better show support values. B) Maximum likelihood based tree obtained with the reduced dataset (this tree was used for the PAML calculations). C) Bayesian analysis on the complete dataset. (PDF) [file pone.0113872.s002.pdf]

Figure S2. **Bayesian and maximum likelihood phylogenetic analysis of the gene for mikimopine synthase and the ORF14 gene in *A. rhizogenes* and their plant homologues in the genera *Nicotiana* and *Linaria.*** The clades belonging to individual plant species are marked in colour. If left and right copy are present they are also distinguished by colour. A) Maximum likelihood based tree displayed as cladogram to better show support values (aLRT). B) Maximum likelihood based tree obtained with the reduced dataset (this tree was used for the PAML calculations). C) Bayesian analysis on the complete dataset.

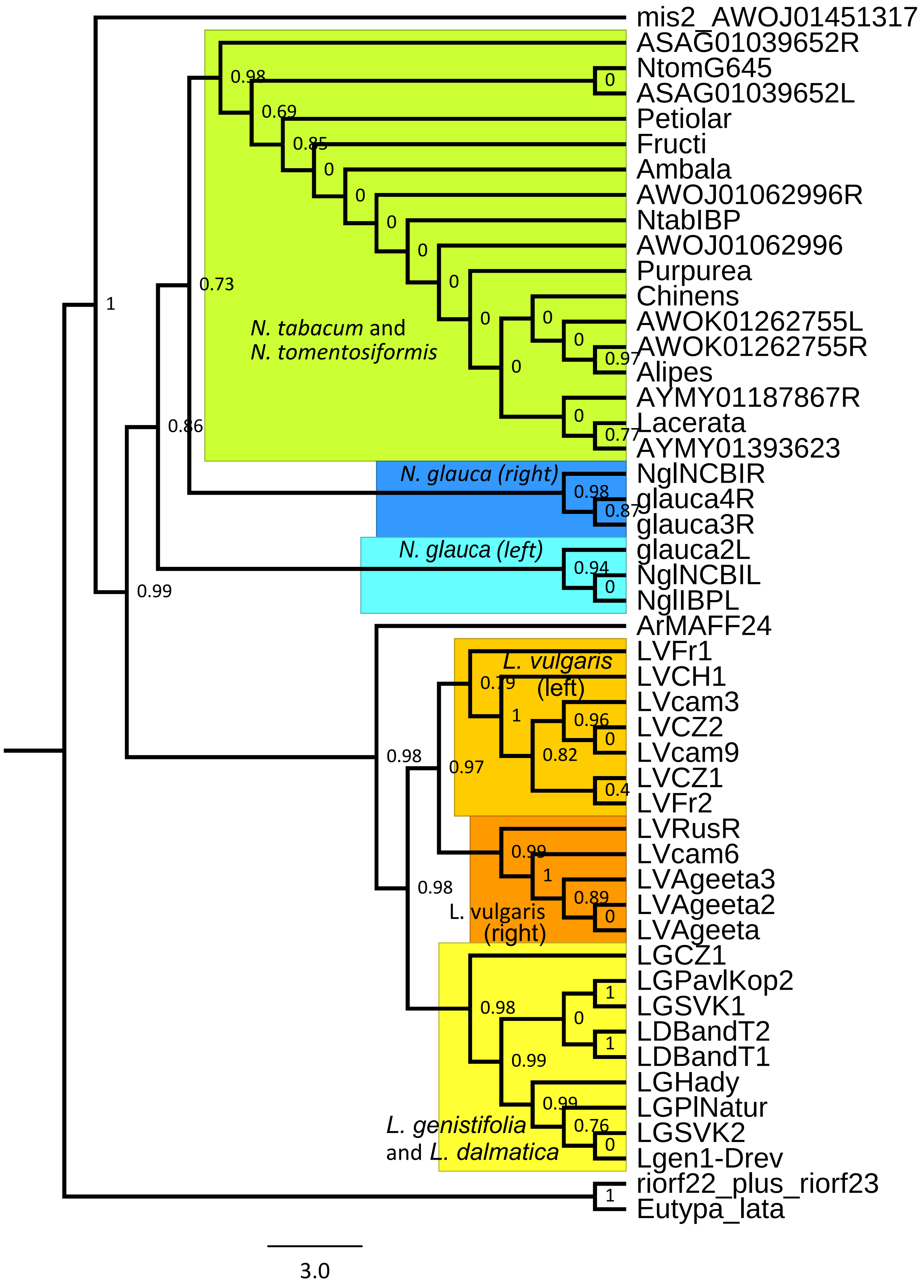

Figure S2A. Maximum likelihood based tree displayed as cladogram to better show support values (aLRT).

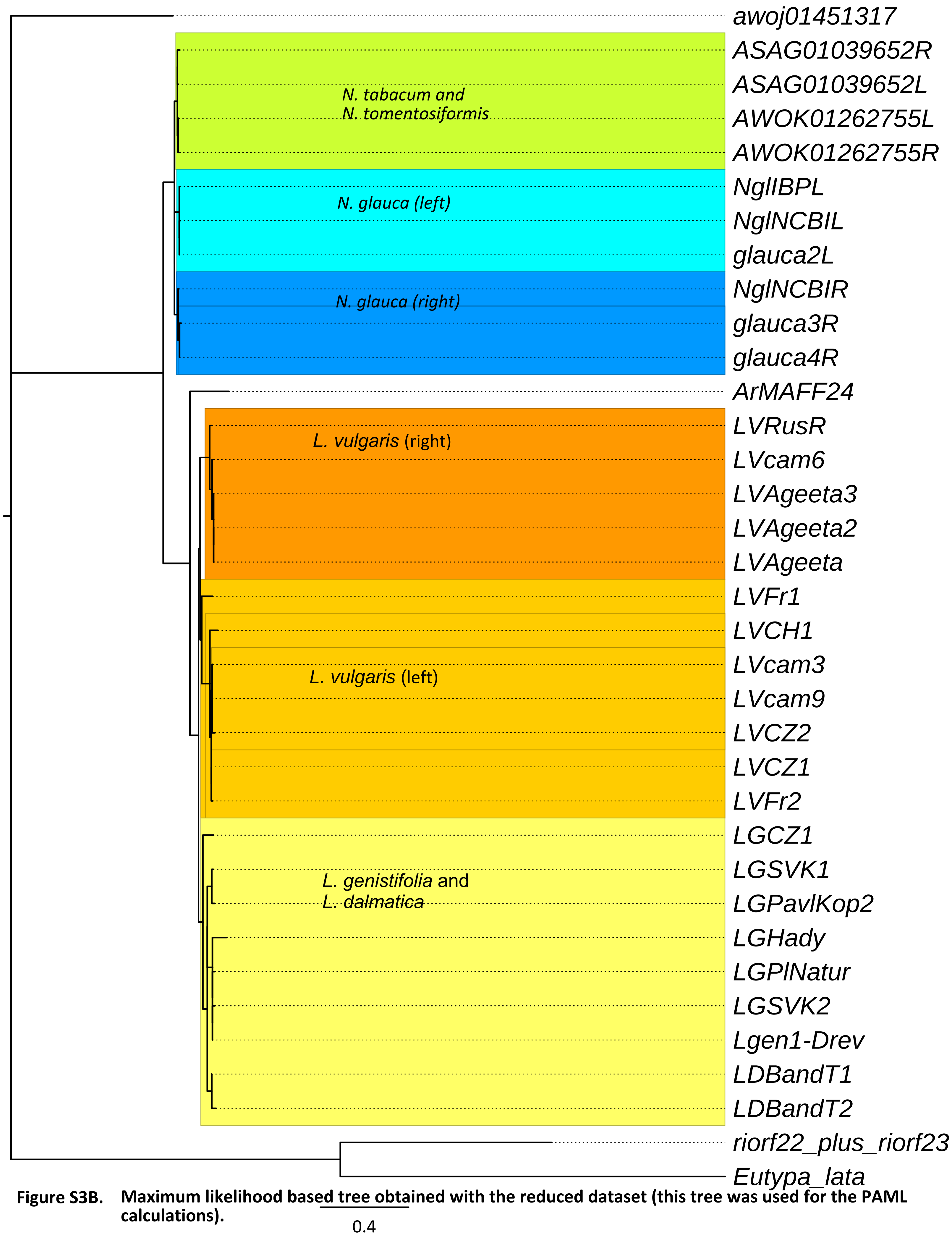

Figure S3B. **Maximum likelihood based tree obtained with the reduced dataset (this tree was used for the PAML calculations).**

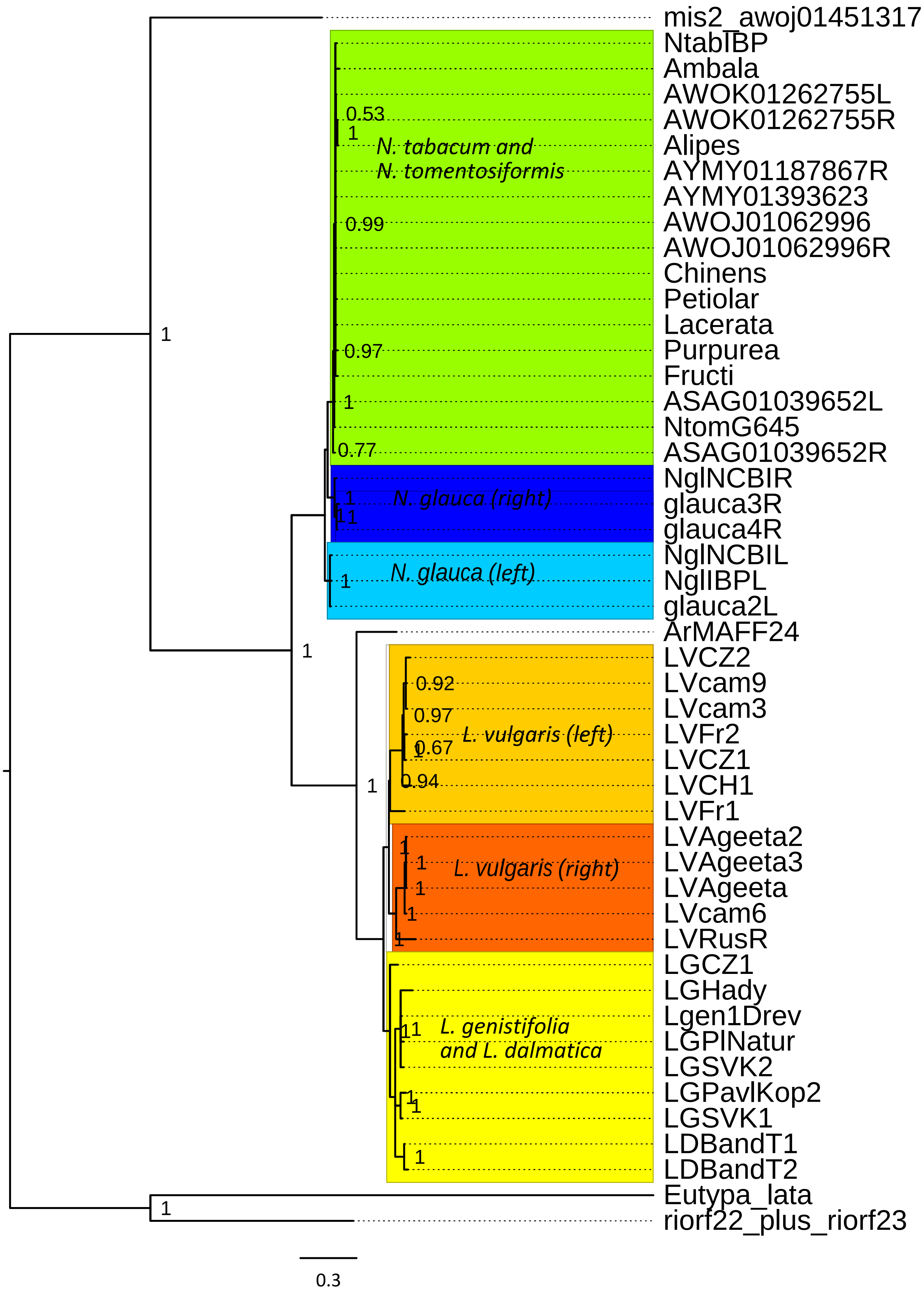

Figure 2C. Bayesian analysis on the complete dataset.
