# Supplementary material for: The Evolutionary Fate of the Horizontally Transferred Agrobacterial Mikimopine Synthase Gene in the Genera Nicotiana and Linaria
Source: PLoS One. 2014 Nov 24;9(11):e113872. doi: 10.1371/journal.pone.0113872 (PMC4242671; doi:10.1371/journal.pone.0113872)

Figure S3. Tests of the monophyletic origin of the *mis* insertion in the genome of *Linaria* and dating of this HGT event. A) The chronogram is based on the analysis of the chloroplast sequence dataset in Beast 1.7.5. The species carrying the mikimopine synthase gene are labelled by the M letters in a grey box. The support values for each node are listed as follows: posterior probability obtained in Beast 1.7.5, posterior probability obtained in MrBayes 3.2, bootstrap value obtained in GARLI 2.0, and bootstrap value obtained in PAUP 4.0. (The posterior probabilities are not shown if they are lower than 0.8 and the bootstrap values are not shown if they are lower than 60.) The horizontal bars represent 95% HPD (highest posterior density) intervals of node ages in million years. B) Phylogenetic tree obtained via maximum likelihood method (GARLI) using nuclear dataset in the genus Linaria. C) Phylogenetic tree obtained via maximum likelihood method (GARLI) using chloroplast dataset in the genus Linaria. D) Comparison of the phylogenetic trees obtained via maximum likelihood method (GARLI) using nuclear or chloroplast dataset using Compare2tree program.

Figure S3A. The chronogram based on the analysis of the chloroplast sequence dataset in Beast 1.7.5. The species carrying the mikimopine synthase gene are labelled by the M letters in a grey box. The support values for each node are listed as follows: posterior probability obtained in Beast 1.7.5, posterior probability obtained in MrBayes 3.2, bootstrap value obtained in GARLI 2.0, and bootstrap value obtained in PAUP 4.0. (The posterior probabilities are not shown if they are lower than 0.8 and the bootstrap values are not shown if they are lower than 60.)

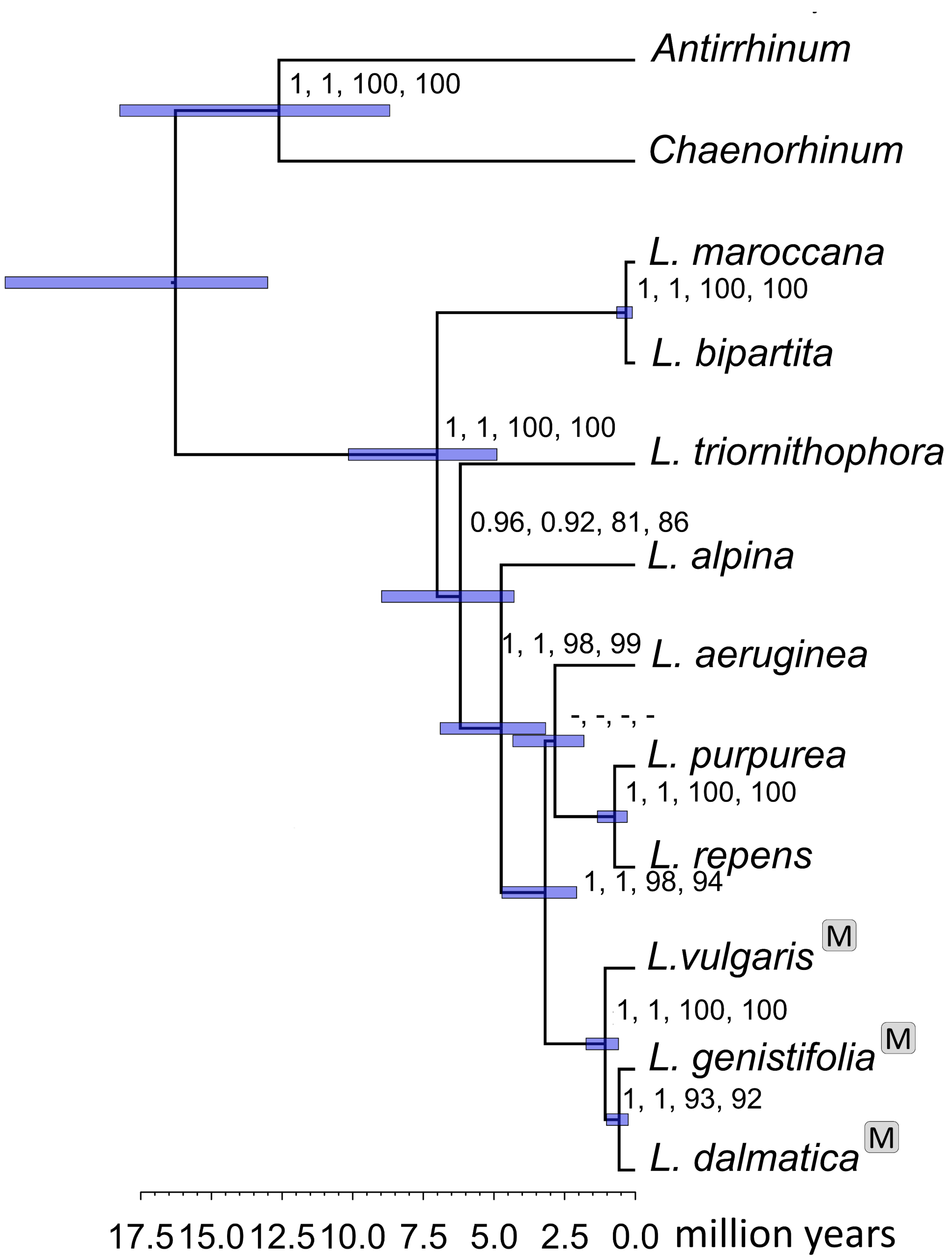

Figure S3B. Phylogenetic tree obtained via maximum likelihood method (GARLI) using nuclear dataset in the genus *Linaria*.

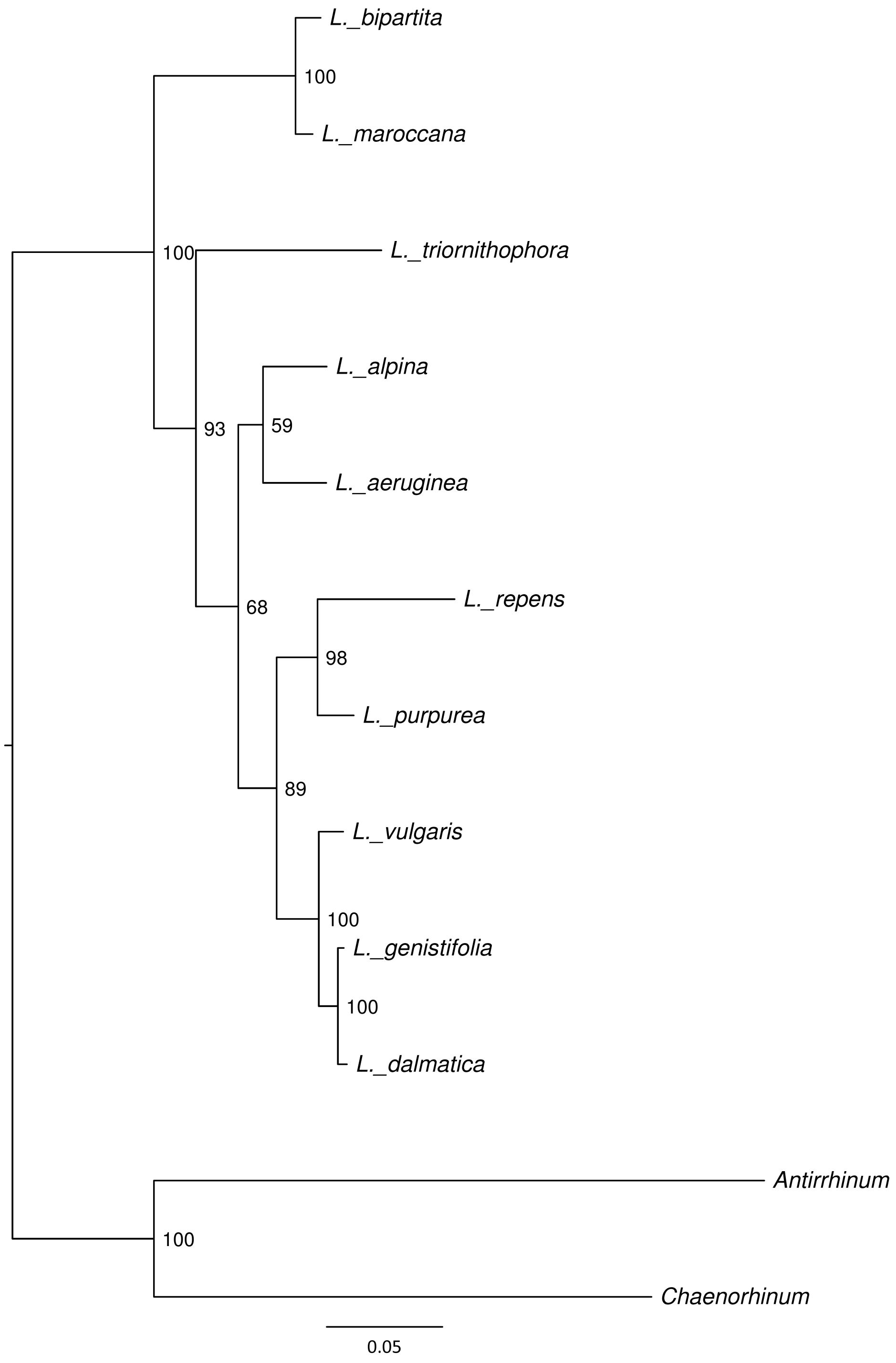

Figure S3C. Phylogenetic tree obtained via maximum likelihood method (GARLI) using chloroplast dataset in the genus *Linaria*.

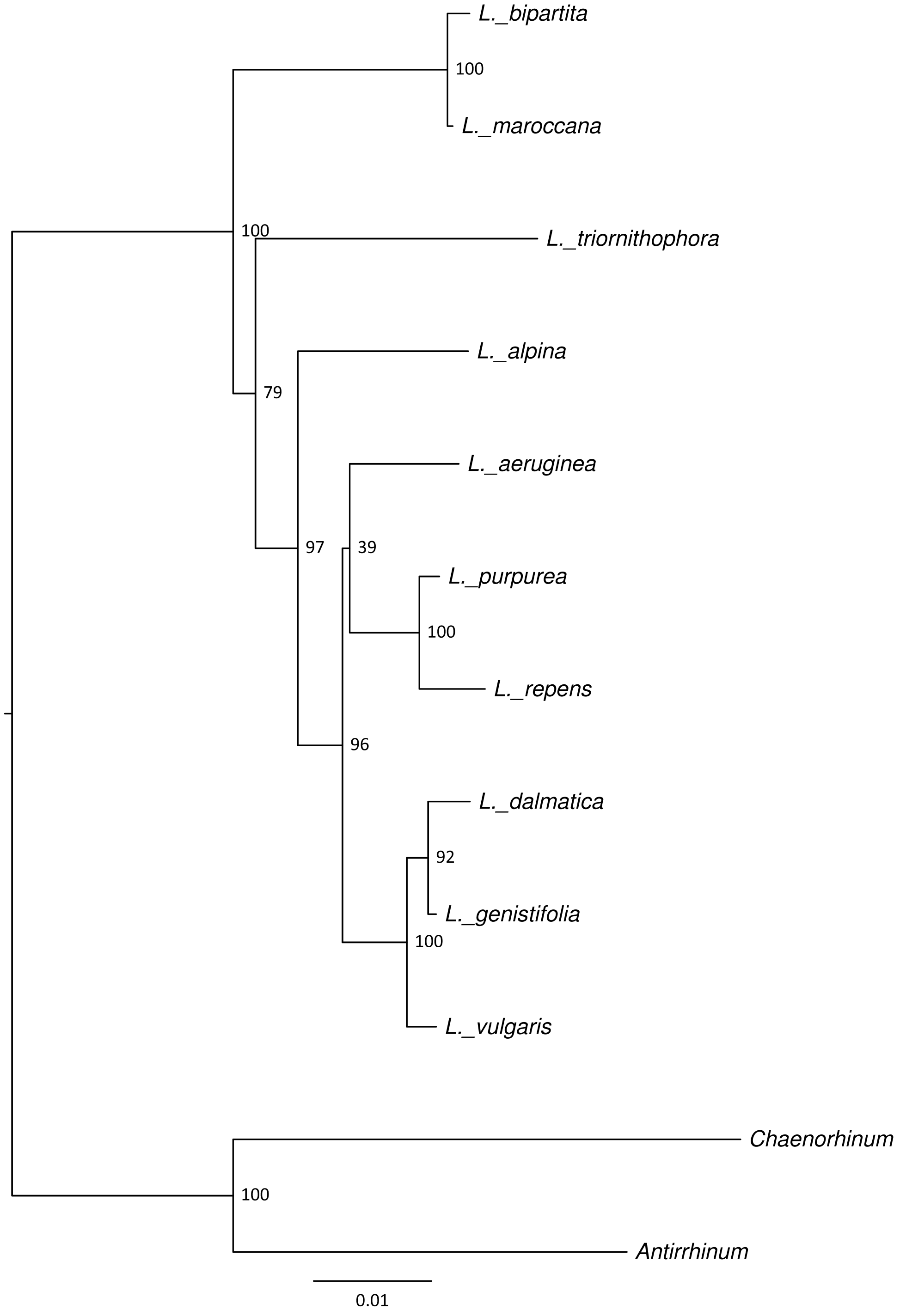

Figure S3D. Comparison of the phylogenetic trees obtained via maximum likelihood method (GARLI) using nuclear dataset (a) or chloroplast dataset (b). The arrows point to the species having different position in the tree based on the chloroplast dataset (b) if compared to tree based on the nuclear dataset (a).

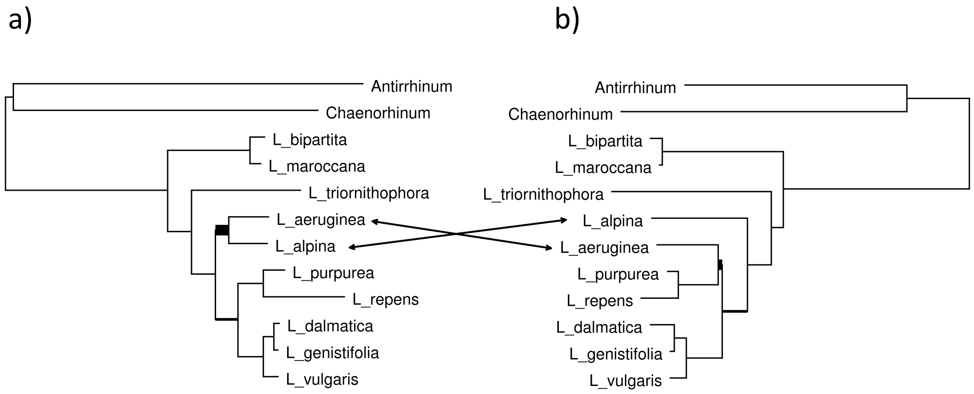

Supplement: Figure S3 — Tests of the monophyletic origin of the mis insertion in the genome of Linaria and dating of this HGT event. A) The chronogram is based on the analysis of the chloroplast sequence dataset in Beast 1.7.5. The species carrying the mikimopine synthase gene are labelled by the M letters in a grey box. The support values for each node are listed as follows: posterior probability obtained in Beast 1.7.5, posterior probability obtained in MrBayes 3.2, bootstrap value obtained in GARLI 2.0, and bootstrap value obtained in PAUP 4.0. (The posterior probabilities are not shown if they are lower than 0.8 and the bootstrap values are not shown if they are lower than 60.) The horizontal bars represent 95% HPD (highest posterior density) intervals of node ages in million years. B) Phylogenetic tree obtained via maximum likelihood method (GARLI) using nuclear dataset in the genus Linaria. C) Phylogenetic tree obtained via maximum likelihood method (GARLI) using chloroplast dataset in the genus Linaria. D) Comparison of the phylogenetic trees obtained via maximum likelihood method (GARLI) using nuclear or chloroplast dataset using Compare2tree program. (PDF) [file pone.0113872.s003.pdf]
