# Supplementary material for: The Evolutionary Fate of the Horizontally Transferred Agrobacterial Mikimopine Synthase Gene in the Genera Nicotiana and Linaria
Source: PLoS One. 2014 Nov 24;9(11):e113872. doi: 10.1371/journal.pone.0113872 (PMC4242671; doi:10.1371/journal.pone.0113872)
Supplement: Figure S4 — Analysis of the putative heterogeneity of the trees sampled in Beast analysis. First 25% trees were removed as burn in. The most frequent topologies and the corresponding consensus trees are shown in blue. The second most frequent topology is shown in red and the corresponding consensus tree is shown in green. A) nuclear dataset – analysis of 20000 trees from Beast 1.7.5 output in DensiTree 2.0.1. B) chloroplast dataset – analysis of 20000 trees from Beast 1.7.5 output in DensiTree 2.0.1. (PDF) [file pone.0113872.s004.pdf]

A

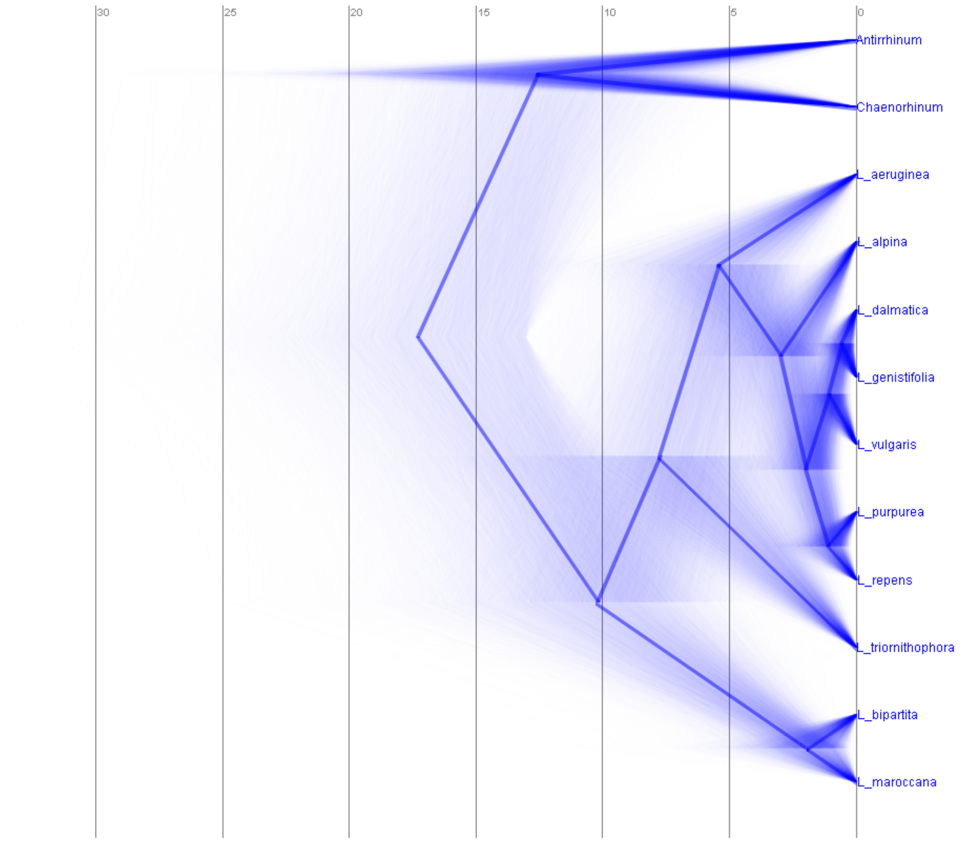

B

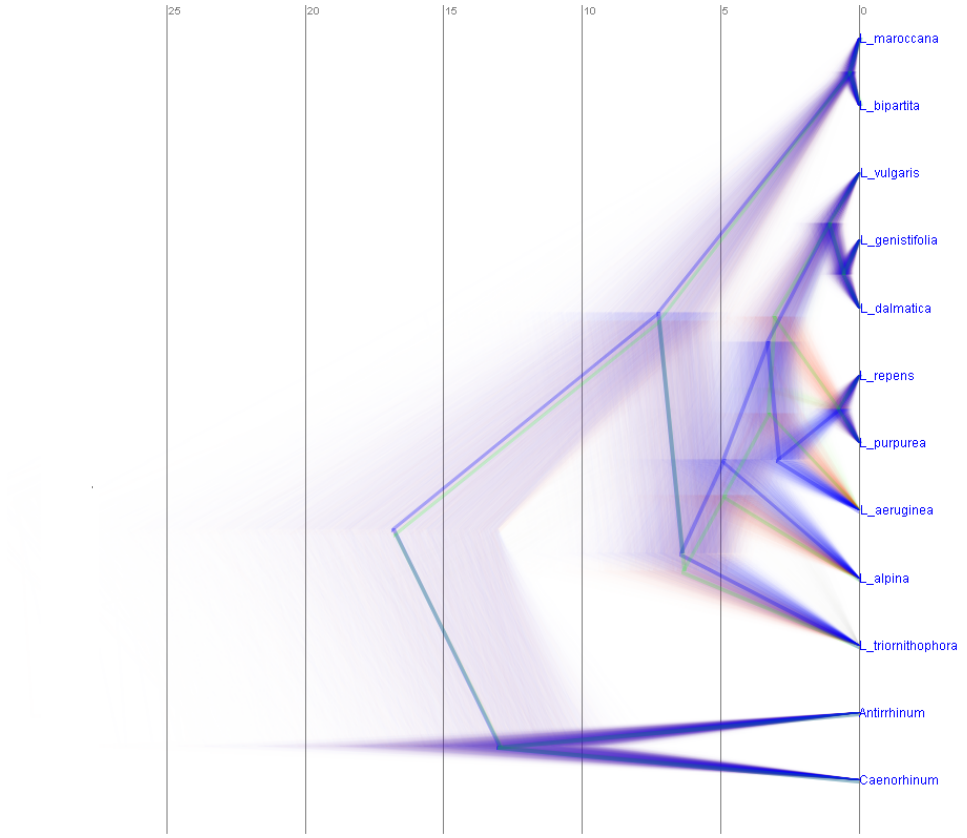

Figure S4. Analysis of the putative heterogeneity of the trees sampled in Beast analysis.
First 25% trees were removed as burn in. The most frequent topologies and the corresponding consensus trees are shown in blue. The second most frequent topology is shown in red and the corresponding consensus tree is shown in green. A) nuclear dataset - analysis of 20000 trees from Beast 1.7.5 output in DensiTree 2.0.1. B) chloroplast dataset - analysis of 20000 trees from Beast 1.7.5 output in DensiTree 2.0.1. First 25% trees were removed as burn in.
