# Supplementary material for: The Evolutionary Fate of the Horizontally Transferred Agrobacterial Mikimopine Synthase Gene in the Genera Nicotiana and Linaria
Source: PLoS One. 2014 Nov 24;9(11):e113872. doi: 10.1371/journal.pone.0113872 (PMC4242671; doi:10.1371/journal.pone.0113872)

Figure S5. Analysis of the coverage of the mikimopine-synthase homologue in N. tabacum by small RNAs as scored in roots (A), stems (B) and leaves (C).

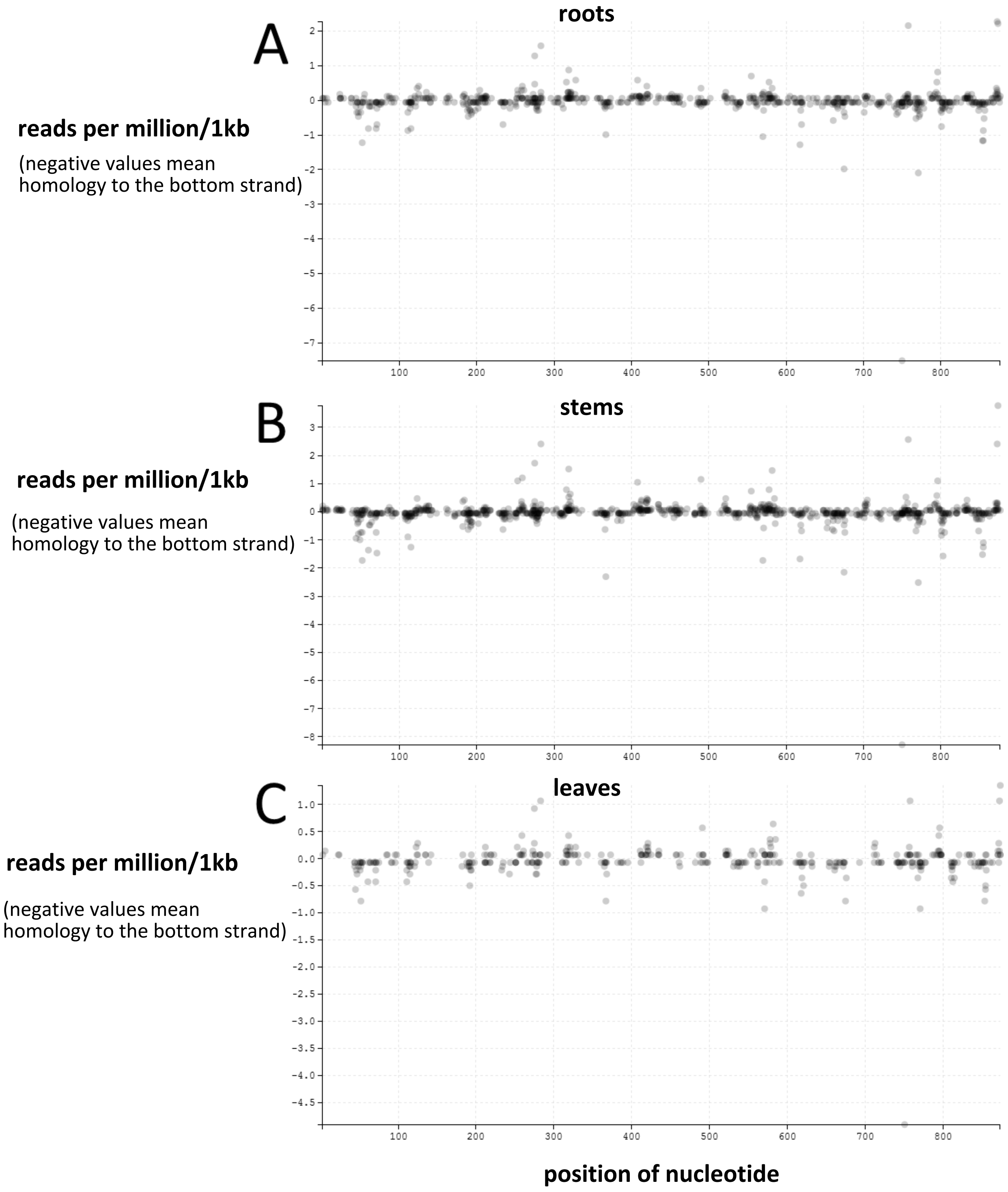

Supplement: Figure S5 — Analyses of the distribution of small RNAs along the mis sequence homolog in N. tabacum. The abundance of reads is shown as number of reads per one kilobase and per million of reads. (PDF) [file pone.0113872.s005.pdf]
