# Supplementary material for: The Evolutionary Fate of the Horizontally Transferred Agrobacterial Mikimopine Synthase Gene in the Genera Nicotiana and Linaria
Source: PLoS One. 2014 Nov 24;9(11):e113872. doi: 10.1371/journal.pone.0113872 (PMC4242671; doi:10.1371/journal.pone.0113872)
Supplement: Figure S6 — Analysis of the abundance of homologous small RNAs for 100 randomly chosen genes in N. tabacum . The abundance of reads is shown as number of reads per one kilobase and per million of reads These results were compared with results obtained in mis. (PDF) [file pone.0113872.s006.pdf]

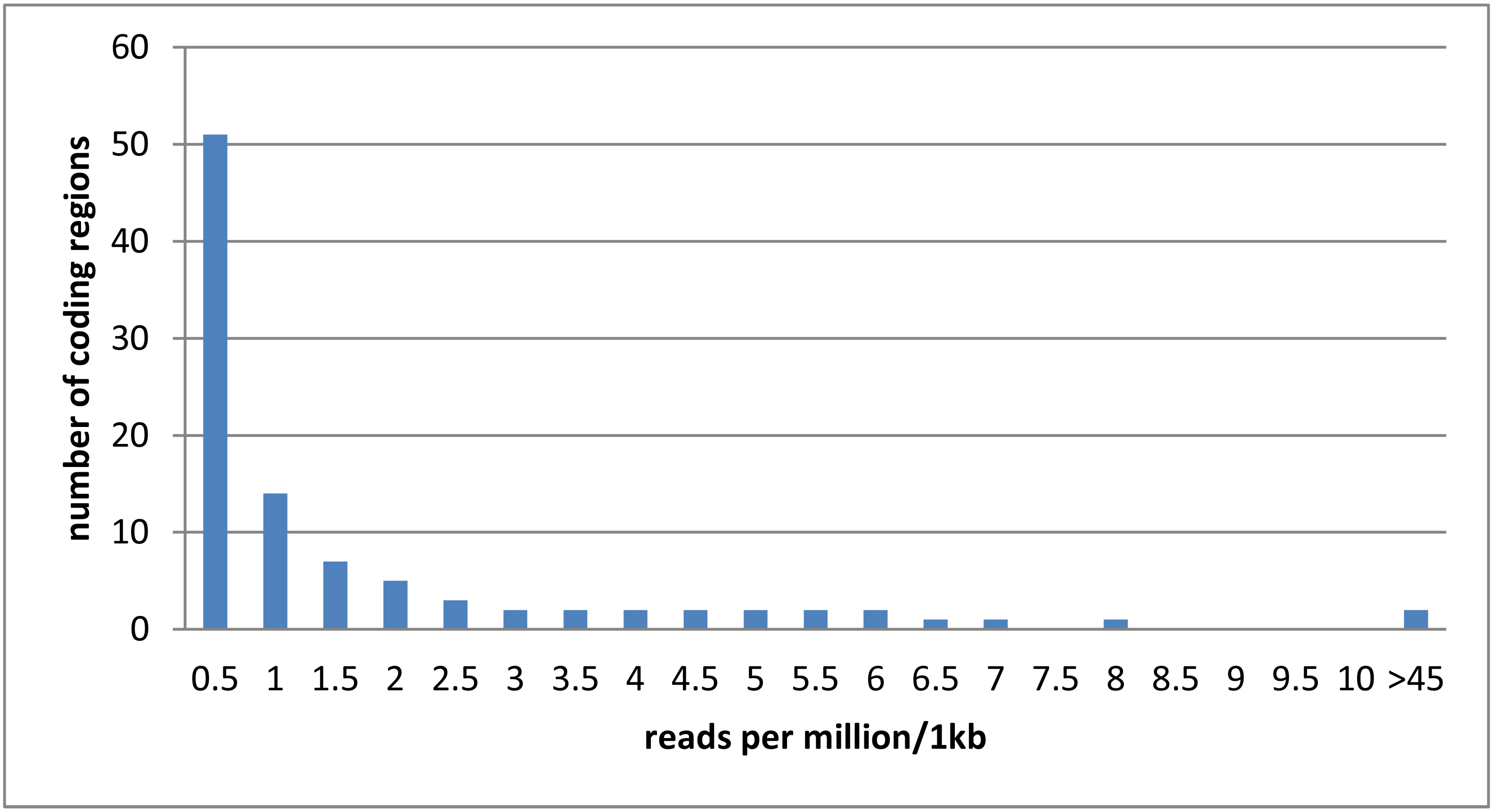

Figure S5. Analyses of the distribution of small RNAs along the mis sequence homolog in *N. tabacum.* The abundance of reads is shown as reads per million.
